# Preparation of Antibacterial Cellulose Paper Using Layer-by-Layer Assembly for Cooked Beef Preservation at Ambient Temperature

**DOI:** 10.3390/polym10010015

**Published:** 2017-12-23

**Authors:** Hui Li, Rongqi Cui, Lincai Peng, Shengbao Cai, Pan Li, Tianqing Lan

**Affiliations:** 1 Research Institute of Food Safety, Kunming University of Science and Technology, Kunming 650600, China; lihuiscut@126.com (H.L.); C18487112191@126.com (R.C.); caicau2009@126.com (S.C.); l15066335173@126.com (P.L.); 2 Faculty of Chemical Engineering, Kunming University of Science and Technology, Kunming 650500, China; penglincai8@163.com

**Keywords:** layer-by-layer, antibacterial paper, tensile strength, ε-poly(l-lysine), cooked beef preservation

## Abstract

Positively-charged ε-poly(l-lysine) (ε-PL) and negatively-charged carboxymethyl cellulose (CMC) were alternately deposited on a cellulose paper surface by the layer-by-layer (LBL) assembly technique. The formation of ε-PL/CMC multilayers was confirmed by X-ray photoelectron spectroscopy (XPS), Fourier transform infrared spectra (FTIR), and zeta potential measurement. The morphologies of the multilayer-modified cellulose paper were observed by scanning electron microscopy (SEM). The ε-PL/CMC multilayers effectively improved not only the antibacterial activity of cellulose paper against both *Escherichia coli* and *Staphylococcus aureus*, but also the cellulose paper tensile strength property. Cellulose paper modified with a (ε-PL/CMC)_4.5_ multilayer exhibited the strongest antibacterial activity, selected for preserving cooked beef for nine days at ambient temperature, could extend the shelf-life of beef for about three days compared with common commercial PE films. The prepared antibacterial paper did not show any evidence of the cytotoxic effect since it could not increase the cytoplasmic lactate dehydrogenase release from L-929 fibroblast cells in contact with the antibacterial paper, suggesting the possibility of utilization in food packaging field.

## 1. Introduction

Today, petroleum-based synthetic plastics, such as polypropylene (PP), polyethylene (PE), and polystyrene (PS), have been widely used to prepare food packaging materials due to their excellent mechanical and barrier properties, ease of processing, and relatively low cost. Unfortunately, these plastic materials are non-biodegradable and, thus, result in serious environmental pollution [1]. Moreover, plasticizers, one of additives in plastics, may migrate from plastic packaging materials into foodstuffs during processing, transport, and storage, causing potential adverse effects on food safety and human health [2]. Therefore, much attention has been paid to the development of biodegradable food packaging materials using green biopolymer to substitute petroleum-based plastic packaging materials [3,4,5].

Cellulose paper produced from cellulosic fibers has been regarded as an inexhaustible and renewable resource [6,7,8]. Owing to its unique characteristics of low cost, biodegradability, recyclability, mechanical flexibility, printability, and affordability, cellulose paper has been successfully used as a packaging materials for food preservation [9,10]. However, natural cellulose fiber-based papers have no antibacterial activity and can only preserve food by prohibiting microbes from contacting food; thus, the shelf life of food cannot be extended long enough [11]. To solve this shortcoming, a number of methods have been employed to impart antibacterial activity to cellulose fiber-based products, including chemical grafting [12], reversible addition-fragmentation chain transfer (RAFT) polymerization [13], atom transfer radical polymerization (ATRP) [14], and radio frequency (RF) co-sputtering coating antibacterial substances onto the cellulose fiber surface [15]. Unfortunately, all these above-mentioned methods suffer from drawbacks, such as uncontrollable grafting yield, tedious and complex reaction procedures, and costly equipment, which limit their wide applications.

The layer-by-layer (LBL) self-assembly technique, introduced first by Decher et al., has become one of the versatile and simple surface modification methods through preparing functional multilayers on the surface of various solid substrates [16,17]. The formation of LBL multilayers is based on the alternate deposition of oppositely-charged species on a charged solid surface by a simple dipping procedure [17]. The constructed LBL multilayers have found uses in many applications, such as bioactive coatings [18], electrically conductive coatings [19], and hollow polyelectrolyte capsules for drug delivery [20]. Recently, numerous studies have been reported concerning the fabrication of antibacterial cellulose fiber-based products by immobilization antibacterial silver nanoparticles (Ag NP) onto fiber surfaces using the LBL technique [21,22,23,24,25]. However, Ag NP is prepared by using the traditional chemical reduction method that requires the use of chemical agents, such as hydrazine hydrate and sodium borohydride, which may cause environmental pollution problem [26]. Moreover, potential toxic effect of Ag NP nanoparticles have been reported in many studies [27,28,29], which restricts their application in the medical and food industries. Therefore, a safe and environmentally-benign antibacterial agent for producing antibacterial cellulose paper is urgently needed [30,31,32,33].

Natural antibacterial agents have attracted considerable interest due to their many unique properties, such as nontoxicity, biodegradability, and easy availability [34,35,36]. Chitosan [37], lysozyme [38], and antibacterial peptides [39] are great candidates as building blocks to prepare LBL multilayers with antibacterial activity for bio-based active packaging [40]. Especially, antibacterial peptides are a class of natural antibacterial substances and have gained considerable attention for food preservation for their excellent antibacterial activity and established safety [41]. ε-Poly(l-lysine) (ε-PL) is a homopolymer of l-lysine through the isopeptide bond between ε-amino and α-carboxyl groups [42]. In contrast to poly(l-lysine), which is synthesized by chemists, ε-PL is a natural cationic biopolymer produced by the bacterium *Streptomyces albulus* [43]. ε-PL is also water-soluble, biodegradable, edible, and has been reported to have a wide antibacterial spectrum against yeasts, molds, and Gram-positive and Gram-negative bacterial species [44,45,46]. Due to its excellent antibacterial activity, heat stability, and nontoxicity toward humans and the environment, ε-PL has been utilized as food preservations additive [47], dietary agent [48], blending materials for antibacterial composite films [49], and it has been previously utilized to introduce antibacterial properties to silk and wool fibers [50,51].

In this work, we presented a facile method to prepare antibacterial cellulose paper by LBL deposition of cationic ε-PL and anionic carboxymethyl cellulose (CMC) on the paper’s surface. The effects of ε-PL/CMC multilayers on the paper’s bacterial inhibition ability and the paper’s physical strength were investigated, respectively. Finally, the obtained antibacterial cellulose paper was applied for cooked beef preservation and the antibacterial performance was evaluated.

## 2. Materials and Methods

### 2.1. Materials

Fully-bleached eucalyptus kraft pulp was kindly provided by Yunnan Yunjing Forestry and Pulp Co., Ltd. (Puer, China). Before use, the pulp was washed, and the carboxyl groups of pulps were converted to their sodium forms according to Marais et al. [52]. ε-poly(l-lysine) (ε-PL)was purchased from Hangzhou Xinyue Biotechnology Co., Ltd. (Hangzhou, China). Carboxymethyl cellulose (CMC) was purchased from Bomei Biotechnology Reagent Co., Ltd. (Hefei, China). All aqueous solutions were prepared using the ultrapure water (Millipore, Bedford, MA, USA) with a resistivity of 18.2 MΩ/cm. In addition, *Escherichia coli* and *Staphylococcus aureus* were obtained from the Research Institute of Food Safety of Kunming University of Science and Technology (Kunming, China). Fresh beef used for preservation testing was purchased from a local supermarket. All other chemical reagents were used directly without further purification.

### 2.2. Preparation of ε-PL/CMC Multilayers on the Cellulose Fiber Surface

**Scheme 1:**
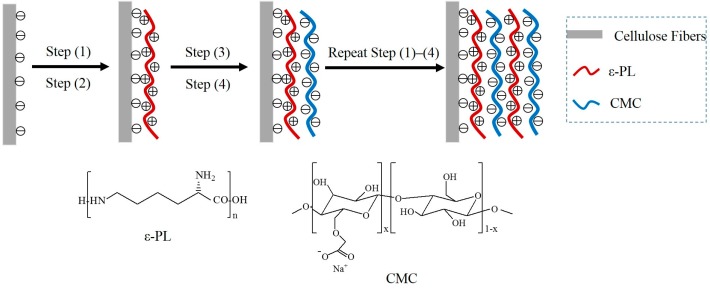
Schematic representation of the preparation process of antibacterial paper.

The preparation process of ε-PL/CMC multilayers on the fiber surface was identical with our previous work [53]. First, cellulose fibers were immersed into positively-charged ε-PL solution (1 mg/mL) for 20 min, followed by rinsing with ultrapure water three times (Steps 1–2). Then, the cellulose fibers were immersed into a negatively-charged CMC solution (1 mg/mL) for 20 min, followed by an identical rinsing procedures (Steps 3–4), as shown in Scheme 1. The procedure was repeated until the desired number of deposition bilayers was obtained. Here, (ε-PL/CMC)*_n_* was used as a formula to label the LBL multilayers, where n was the number of the ε-PL/CMC bilayers.

### 2.3. Characterizations

The surface element composition of the samples was investigated by X-ray photoelectron spectroscopy (XPS) by using an Axis Ultra System DLD spectrometer (Kratos Analytical Ltd., Manchester, UK) with Al *K*α radiation. Survey spectra were recorded for the 0–1350 eV binding energy range. The surface chemical groups of the samples was measured by attenuated total reflection-Fourier transform infrared spectroscopy (ATR-FTIR; Magna-IR 750 spectrometer, Nicolet Instrument, Thermo Company, Waltham, MA, USA). The surface potential of the samples was examined by using a Mütek SZP-10 zeta potential tester (BTG Group, Herrsching, Germany) based on the streaming potential method. The surface morphology of the samples was observed by scanning electron microscopy (SEM, Hitachi S-4800, Tokyo, Japan). The samples for SEM observations were dried at room temperature, and sputter-coated with gold before observation.

### 2.4. Antibacterial Test

The antibacterial assay was performed according to the shaking flask method reported by Qian et al. [54]. A 0.1 g paper sample was placed in a flask containing 5 mL of bacterial suspension (10^6^ CFU/mL). After shaking at 200 rpm at 37 °C for 3 h, 1 mL of bacterial suspension was successively diluted with physiological saline into different test tubes. Then, 0.1 mL of the resulting diluted bacterial culture was placed onto LB agar in a Petri dish. The plates were incubated at 37 °C for 24 h and the number of viable bacteria was counted. Three repeats were carried out for each sample. The growth inhibition of bacteria can be quantified by the following equation:

$$\mathrm{Growth}\;\mathrm{Inhibition}\;\mathrm{Degree}\;\mathrm{of}\;\mathrm{Bacteria}=\frac{A_{0}-A}{A_{0}}\times 100\%$$

where *A*_0_ and *A* are the number of the colonies of the control and tested samples, respectively.

### 2.5. Paper Physical Strength Test

Prior to the paper strength measurement, the cellulose fibers were first made into paper handsheets with a grammage of 80 g/m^2^ using a semiautomatic sheet former equipped with a circulation water system. After drying, the handsheets were kept in a constant temperature and humidity room (23 °C, 50% relative humidity) for 24 h, then cut into 15 mm wide strips. The tensile strength of the paper samples were measured using a tensile tester (DCP-KZ300, Sichuan Changjiang Paper Instrument Co., Yibin, China). The gauge length is 10 cm. The values presented were the y7 average from at least five measurements for each sample. The zero-span tensile strength was determined using a Pulmac zero-span tensile tester (Pulmac International Inc., Middlesex, CT, USA) according to the ISO 15361:2000 standard.

### 2.6. Antibacterial Paper Applied for Cook Beef Preservation

The beef was trimmed of visible fact, connective tissue and adhering skin before using. A total of 1200 g of beef was cut into 60 equally-small cubes by weight. These small beef cubes were cooked for 15 min at 90 °C in a water bath, and then divided into four parts as three control groups and a test group, respectively. The test group was packaged with antibacterial paper bags while the other three control groups were packaged with no film, PE film, and original paper, respectively. The samples were placed under ambient temperature (23 °C) and taken out after 0, 3, 6, and 9 days of storage for the determination of microbial and physicochemical properties.

For microbial analysis: The sample was homogenized with 100 mL of sterile 0.1% peptone water for 3 min at room temperature. For each sample, appropriate serial dilutions were prepared in sterile saline and 100 μL appropriate dilution was spread on plate count agar in a Petri dish. The plates were incubated at 37 °C for 48 h and the total number of bacteria in cooked beef were determined by counting the number of colony-forming units. The total number of bacteria (CFU/g) were log-transformed. The experiment was performed in triplicate.

For physicochemical properties analysis: The sample was homogenized with 100 mL non-ammonia purified water for 2 min and impregnated for 30 min with interval stirring every 5 min. Then the resultant solution was filtered to obtain a cooked beef infusion. The pH of the infusion was measured by a digital pH meter (Mettler-Toledo Co., Ltd., Greifensee, Switzerland). The total volatile basic nitrogen (TVB-N) in cooked beef was measured by stream distillation method [55].

### 2.7. Cytotoxicity Assay

The cytotoxicity of the prepared antibacterial paper was performed according to the method reported by Lee et al. [56] with some modifications. The released amounts of the cytoplasmic lactate dehydrogenase (LDH) from the cells incubated by the antibacterial paper were determined. First, 500 μL L-929 fibroblast cell suspensions with a density of approx. 2 × 10^4^ cells/mL were added to 24-well culture plate and incubated at 37 °C for 24 h, then cell culture medium was aspirated and 1 mL fresh cell culture medium was added to each well in culture plate. Subsequently, the sterilized paper specimen with an area of 1 × 1 cm was immersed into the cell culture medium and incubated at 37 °C.

After a preset incubation time, the medium was taken out from each well and centrifuged at 350 g for 5 min, and the supernatant was mixed with the reagents in an LDH assay kit (Nanjing Jiancheng Bioengineering Institue, Nanjing, China). The absorbance of the reaction mixture was measured using a microplate reader at 450 nm. The released amounts of LDH from cells cultured on a 24-well plate without an immersion paper specimen were used as the control groups, whereas the released amounts of LDH from the cells lysed with 1% Triton X-100 were used as positive control groups. The cytotoxicity of the tested sample was calculated by the following equation:

$$\mathrm{Cytotoxicity}\;(\%)=\frac{{\mathrm{LDH}}_{T}}{{\mathrm{LDH}}_{P}}$$

where LDH_T_ represents the released amounts of LDH from the tested samples, and LDH_P_ represents the released amounts of LDH from the positive control group.

## 3. Results and Discussion

### 3.1. The Formation of ε-PL/CMC Multilayers on Cellulose Fiber Surfaces

**Figure 1:**
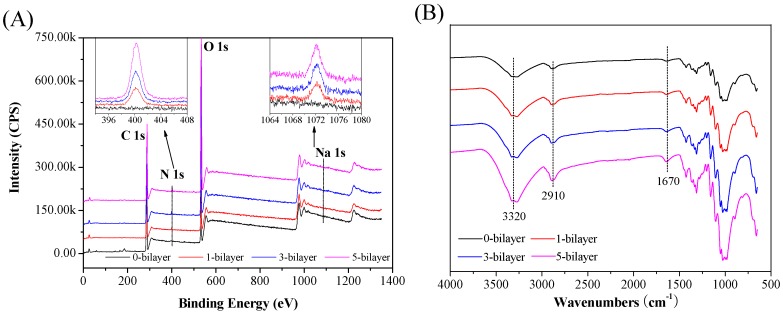

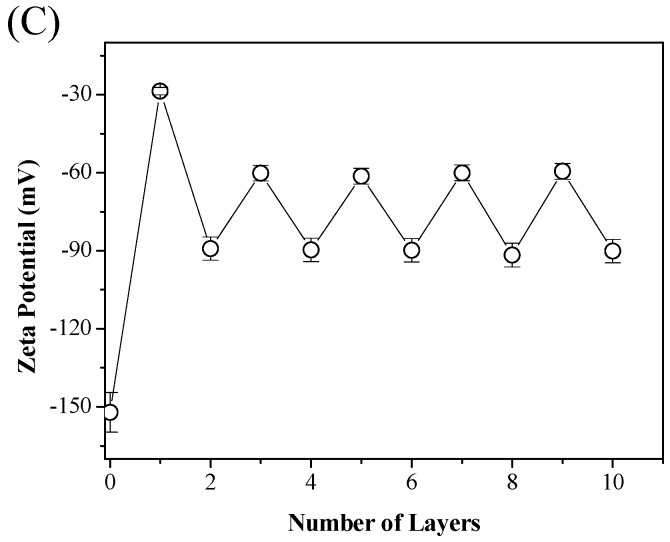
(**A**) X-ray photoelectron spectroscopy (XPS) spectra of original and modified paper samples, with the insets showing high-resolution XPS spectra of N 1s and Na 1s region, respectively; (**B**) FTIR spectra for different paper samples; and (**C**) the zeta potential of the original and modified cellulose fibers as a function of the number of layers.

XPS is a surface-sensitive analysis technique, which is capable of providing both qualitative and quantitative information about the presence of different elements at the surface. Figure 1A shows the qualitative XPS survey spectra of original and ε-PL/CMC multilayer-modified cellulose fibers. The original cellulose fiber has two obvious peaks, the binding energies are 284.7 eV for C, and 533.1 eV for O. Two additional new elements, N (binding energy at 400.2 eV) and Na (binding energy at 1072.2 eV), are observed for ε-PL/CMC multilayer-modified cellulose fibers when compared with the original cellulose fiber. Nitrogen originates from the abundant amino and amide groups present in the ε-PL molecule, and the existence of sodium is attributed to the CMC molecule. Therefore, these two characteristic elements could be used to indicate the multilayer growth on cellulose fibers. Insets in Figure 1A show the high-resolution XPS spectra for N and Na regions for samples with various deposition bilayers, respectively. The peak intensities of N and Na increased with the increasing bilayer number, suggesting the formation of ε-PL/CMC multilayers on fiber surfaces. 

In order to further monitor the LBL growth of ε-PL/CMC multilayers on fiber surface, the ATR-FTIR spectra of the original and ε-PL/CMC multilayer-modified papers are shown in Figure 1B. The original paper shows characteristic peaks of cellulose, which contains the O–H absorption peak at 3320 cm^−1^, C–H absorption peak at 2910 cm^−1^, and C–O–C absorption band from 950 to 1200 cm^−1^. With the deposition of ε-PL/CMC multilayers, these characteristic peak intensities increased due to the presence of CMC, which has a similar sugar unit structure with cellulose. Moreover, a new signal at 1670 cm^−1^ was observed in the spectra of modified paper samples, which was mainly assigned to the C=O stretching vibration in the amide groups of ε-PL.

The step-wise deposition of ε-PL and CMC on cellulose fiber was also monitored by measuring the zeta potential upon addition of each polymer layer. The zeta potential of the modified cellulose fibers versus the deposited layers is shown in Figure 1C. The original cellulose fibers has a negative potential of −152 mV. Subsequently, the regular alternative switch of zeta potential was observed as the LBL assembly proceeded; the successful deposition of the ε-PL/CMC multilayer on the cellulose fiber surface further was, therefore, confirmed.

**Figure 2:**
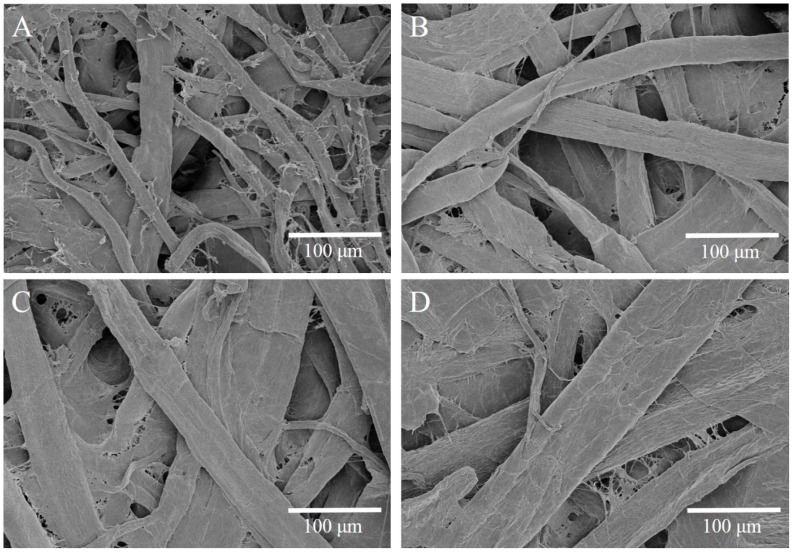
SEM images for different paper samples: (**A**) original paper; (**B**)‒(**D**) paper modified with a (ε-PL/CMC)_1_, (ε-PL/CMC)_3_ , and (ε-PL/CMC)_5_ multilayers, respectively.

SEM was employed to observe the surface morphological changes for paper samples after the ε-PL/CMC multilayers deposition, and the representative SEM images are provided in Figure 2. It can be seen that the original paper consisting of cellulose fibers has a looser and porous surface structure. However, after the deposition of ε-PL/CMC multilayers, some gelatinous substances appeared and resulted in bridging between adjacent cellulose fibers. With the increasing number of bilayers, the average diameters of the cellulose fibers increased, and more gelatinous substances were presented, hence causing more bridging and bonding between fibers, resulting in a more compact surface structure. This observation was consistent with the results reported by Wu and Farnood [57], who studied a CMC/chitosan system on cellulose fiber surfaces. The observed results suggested that ε-PL and CMC forms gels during the LBL deposition, which is highly efficient in improving fiber-fiber bonding, and is ultimately beneficial to the strength properties of cellulose fiber networks (i.e., paper). Further details regarding this, will be discussed in the following section.

### 3.2. Antibacterial Activities of Modified Cellulose Paper

**Figure 3:**
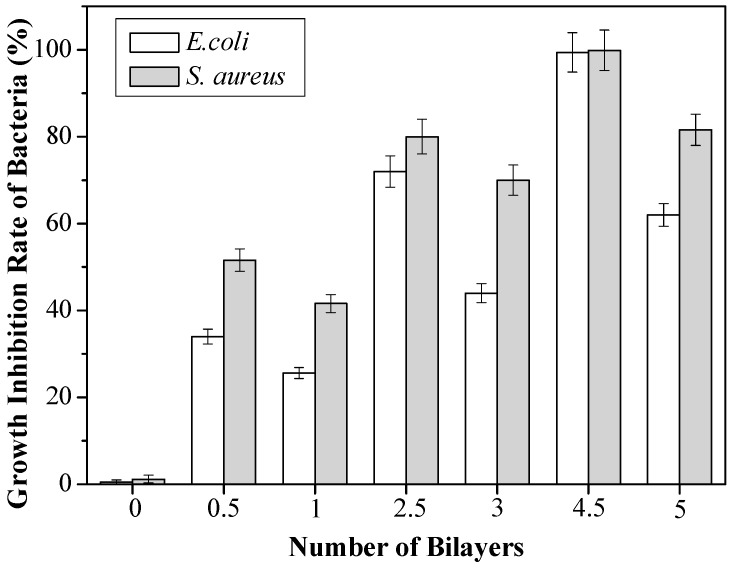
The bacteria growth inhibition rate of paper modified with ε-PL/CMC multilayers having different bilayer numbers.

It has been shown that ε-PL is able to be absorbed onto the bacterial surface, causing stripping of the outer membrane, abnormal distribution of the cytoplasm and, finally, death of the bacteria [42]. Thus, we explored the antibacterial activities of the ε-PL/CMC multilayer-modified paper, and the Gram-negative *E. coli* and Gram-positive *S. aureus* were chosen as model bacteria for the antibacterial activity investigation. The growth inhibition degree of the original and ε-PL/CMC multilayer-modified papers against *E. coli* and *S. aureus* was examined and the results are shown in Figure 3. Apparently, the original paper hardly showed an inhibitory effect, whereas ε-PL/CMC multilayer-modified papers exhibited significant antibacterial activity because of the presence of ε-PL. For all the ε-PL/CMC multilayer-modified paper samples, the inhibition ability against *S. aureus* was better in comparison with that of against *E. coli*. This is because lipopolysaccharides layers of Gram-negative *E. coli* were thicker than that of Gram-positive *S. aureus*, which could protect the bacterial cell wall to from being destroyed by ε-PL. Moreover, it can be observed that the antibacterial activity enhanced with increasing bilayer numbers with either ε-PL or CMC in the outermost layer. Nevertheless, the antibacterial activity of the paper samples with ε-PL in the outermost layer was better than that of the paper samples with CMC in the outermost layer. The growth inhibition degree of (ε-PL/CMC)_4.5_ multilayer-modified paper against *E. coli* and *S. aureus* reach up to 99.4% and 99.9%, respectively, while the growth inhibition degree of (ε-PL/CMC)_5_ multilayer-modified paper against *E. coli* and *S. aureus* decreased to 62.1% and 81.6%. This appearance may be explained by the fact that highly positively-charged ε-PL easily adsorbs negatively-charged bacteria, thus causing efficient contact and interaction with bacteria, but negatively-charged CMC has no antibacterial activity.

### 3.3. Tensile Strength of Modified Cellulose Paper

**Figure 4:**
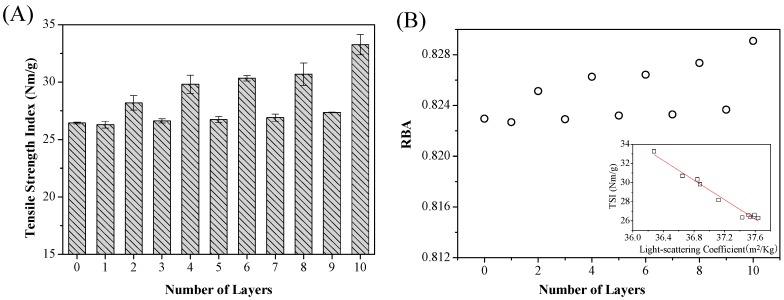
(**A**) Tensile strength index of different paper samples versus the layers number; and (**B**) the relative bonded area (RBA) of different paper samples versus the number of layers.

The influence of ε-PL/CMC multilayers on the paper’s physical strength were also evaluated, and the results are illustrated in Figure 4A. It is clearly shown that paper tensile strength strongly depended on the component in the outermost layer of the ε-PL/CMC multilayers. The tensile strength was significantly higher when CMC was deposited in the outermost layer as compared to when ε-PL was in the outermost layer. The tensile strength index only increased by 3.5% compared with that of the original paper when a 4.5-bilayer (nine-layer) ε-PL/CMC multilayer was deposited on the paper surface, but increased by 25.8% compared with the original paper when a five-bilayer (10-layer) ε-PL/CMC multilayer was deposited. The explanation for this result is that the CMC has a positive effect on the paper strength because it can be used as a strengthening additive in the papermaking industry, which is beneficial to bond between fibers. Bonding ability between fibers that can be indicated by the relative bonded area (RBA) affects the paper tensile strength highly. The RBA was calculated according to our previous method [58], and the RBA for different paper samples are presented in Figure 4B. The RBA was higher when CMC was in the outermost layer than when ε-PL was in the outermost layer, and this tendency is consistent with that of the tensile strength index, demonstrating that the improved paper strength can be mainly attributed to the increase in RBA between fibers. This finding is also supported by the description in the above-mentioned SEM analysis.

### 3.4. Application of Antibacterial Cellulose Paper Packaging on Cooked Beef

**Figure 5:**
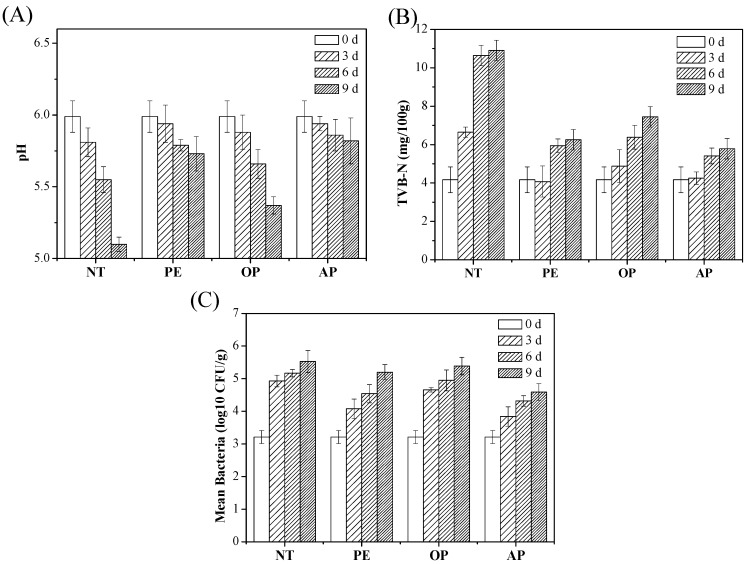
(**A**) pH value; (**B**) total volatile basic nitrogen (TVB-N); and (**C**) total number of bacteria of control group (no treatment, NT), control group (PE film), control group (original paper, OP), and test group (antibacterial paper, AP) in cooked beef during the nine day storage period.

Figure 5 shows the antibacterial performance of the as-prepared antibacterial paper (i.e., paper made from cellulose fibers modified with a 4.5-bilayer ε-PL/CMC multilayer) used as packaging materials for cooked beef, and the control comparison between no treatment (NT), PE, and original paper (OP) is also shown. Figure 5A shows the results of pH variation during a nine-day storage period. Obviously, all the pH values in the beef samples decreased gradually with the storage time, which was probably caused by the growth of lactic acid and other aerobic bacteria which are predominant on cooked meat products [59]. The pH in NT groups dramatically decreased from 5.99 to 5.10 over the nine-day storage period in atmospheric air. Compared with the results of the NT group, a slow decreasing trend was observed in the PE, OP, and AP groups because these packaging materials prevented bacteria in the surrounding atmospheric air from contacting the beef. Especially, the pH of the AP group changed more slightly than the other three control groups, which is attributed to the bacterial inhibition activity of ε-PL.

The TVB-N value, an indicator of spoilage, is mainly composed of ammonia salt by microbial actions and decomposition [60]. Figure 5B shows the changes of TVB-N in cooked beef during storage. It can be seen that the TVB-N value of all the samples increased gradually during the storage period, and the TVB-N value of the AP groups were lower than other three control groups. The total number of bacteria in cooked beef packaged with different materials during the storage period is presented in Figure 5C. It was obvious that the total number of bacteria of all the samples gradually increased with the increasing storage time. On the third day, the total number of bacteria of the NT group increased from 3.21 to 4.93, which had exceeded the limitation of the Chinese hygienic standard for cooked meat (<4.90), whereas the total number of bacteria of the AP group was still 4.59 on the ninth day. Moreover, it was found that the total number of bacteria of the AP groups on the ninth day was similar to that of PE groups on the sixth day (4.54), indicating that antibacterial paper prepared in this study could effectively kill the bacteria on the cooked beef and extend the shelf-life by at least three days compared with PE films, and these results were also confirmed by the TVB-N values.

### 3.5. Cytotoxicity Assay of Preperaed Antibacterial Cellulose Paper

**Figure 6:**
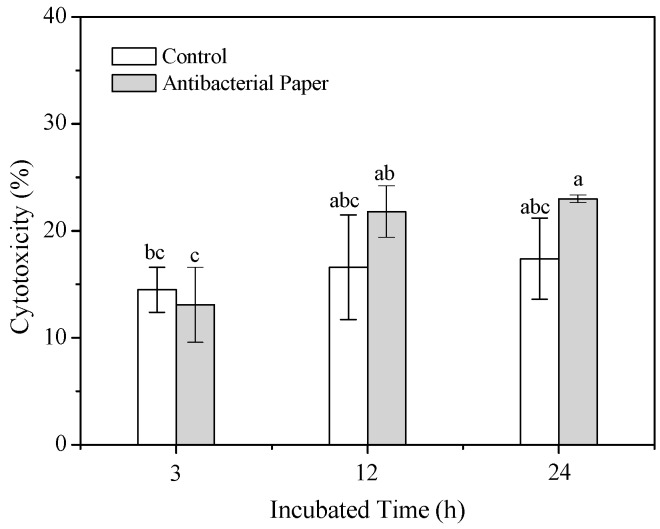
Cytotoxicity of as-prepared antibacterial paper. Values are expressed as the mean ± standard deviation, *n* = 6. Statistical analysis were performed by using the SPSS 22.0 software package (IBM Corporation, Armonk, NY, USA) and the significant differences were determined using one-way analysis of variance followed by Ducan’s multiple range test with a significance level *p* < 0.05.

Since the ultimate objective of our work is to use as-prepared antibacterial cellulose paper as food packaging materials, its cytotoxicity has to be considered first. The released amounts of LDH from cells incubated with a test sample is a good indicator for material cytotoxicity. The cytotoxicity assay results for as-prepared antibacterial paper are shown in Figure 6. The cytotoxicity was less than 23% after 3, 12, and 24 h of direct contact by L-929 cells with the as-prepared antibacterial paper, and there was no significant difference in the released amounts of LDH compared with the control group (*p* > 0.05), demonstrating that as-prepared antibacterial paper was non-toxic to the cell.

## 4. Conclusions

In this study, antibacterial cellulose paper was fabricated by constructing multilayers composed of ε-PL and CMC on a cellulose fiber surface via layer-by-layer assembly. XPS, FTIR, zeta potential, and SEM were employed to validate the formation of ε-PL/CMC multilayers on cellulose fibers. The antibacterial activity testing results showed that ε-PL/CMC multilayers effectively improved the antibacterial activity of cellulose paper, and the antibacterial activity was higher with ε-PL in the outermost layer than that with CMC in outermost layer. The growth inhibition degree of cellulose paper modified with a (ε-PL/CMC)_4.5_ multilayer against *E. coli* and *S. aureus* could reach up to 99.4% and 99.9%, respectively. Additionally, the tensile strength of the cellulose paper enhanced after the deposition of ε-PL/CMC multilayers, and there was a 25.8% increase in tensile strength of cellulose paper modified with the (ε-PL/CMC)_5_ multilayer compared with that of original cellulose paper. Moreover, the cellulose paper modified with a (ε-PL/CMC)_4.5_ multilayer with the best antibacterial activity was selected for cooked beef preservation at ambient temperature, and the results demonstrated that the obtained antibacterial cellulose paper could extend the shelf-life of cooked beef for about three days at ambient temperature. The cytotoxicity assay results indicated the as-prepared antibacterial paper was non-toxic to L-929 cells. Overall, the obtained antibacterial cellulose paper has excellent antibacterial properties and improved tensile strength, which could have a potential application value for commercial applications in the preservation of cooked beef.
